# Transoral Styloidectomy Approach: A Systematic Review and Enhanced Endoscopic Approach

**DOI:** 10.1002/hed.70170

**Published:** 2026-01-16

**Authors:** Nana‐Hawwa Abdul‐Rahman, Vanessa Helou, Lauren A. Gardiner, Paul A. Gardner, Carl H. Snyderman

**Affiliations:** ^1^ Department of Otolaryngology University of Pittsburgh School of Medicine Pittsburgh Pennsylvania USA; ^2^ Department of Neurological Surgery University of Pittsburgh School of Medicine Pittsburgh Pennsylvania USA

**Keywords:** Eagle's syndrome, styloidectomy, systematic review, transoral approach

## Abstract

**Background:**

Synthesize transoral styloidectomy approaches, highlight advantages, disadvantages, surgical outcomes, and describe an improved endoscopic transoral technique.

**Methods:**

A systematic review of peer‐reviewed articles was conducted on November 11, 2025 in PubMed, Embase, the Cochrane Library, and Medline.

**Results:**

Of the 204 articles screened, 45 met inclusion criteria. Four transoral approaches and four visual enhancement techniques were described. Surgical success rate was 94% with no intraoperative complications and a postoperative complication rate of 6.3%. The length of resected styloid averaged 2.91 ± 1.33 cm (range: 1–6 cm). Mean operative time was 47 ± 22 min. Median follow‐up time was 6 months (IQR: 3–12 months).

**Conclusion:**

Transoral styloidectomy is safe and effective for the treatment of Eagle's syndrome. An enhanced endoscopic approach with indocyanine green (ICG) fluoroscopy and neuromonitoring improves intraoperative visualization and helps identify critical vascular structures, potentially reducing the risk of inadvertent injury.

## 1 Introduction

Eagle's syndrome, first described by otolaryngologist Watt Weems Eagle in 1937, is a constellation of neuropathic and vascular occlusive symptoms resulting from pathologic elongation or calcification of the styloid process and/or stylohyoid ligament [1, 2]. This elongation or calcification may be congenital or acquired secondary to trauma (e.g., post‐tonsillectomy, contact sport injuries, and motor vehicle accident) [1, 3]. Accurate diagnosis relies on clinical presentation and physical examination and is confirmed by radiologic imaging. Additional diagnostic methods, including the lidocaine infiltration test and orthopantomography, have also been described [4].

Treatment options for Eagle's syndrome include both medical and surgical intervention, with a reported cure rate of 64.3% and 91.8%, respectively [5]. The two primary surgical approaches are transoral and extraoral. The transoral approach avoids external dissection, thereby minimizing visible scarring and facilitating faster recovery [5]. However, compared to the extraoral approach, it limits surgical site exposure and visualization, which may increase the risk of complications from injury to critical neurovascular structures such as the internal carotid artery (ICA) and cranial nerves. In recent years, various advancements in the transoral approach have emerged to include endoscopic, robotic, and navigation‐guided techniques that have enhanced intraoperative visualization and improved surgical precision [5].

To our knowledge, no systematic review has yet synthesized the various transoral approaches and their associated outcomes. While two systematic reviews by Keirns et al. and Campisi et al. have reported outcomes for transoral robotic surgery, this represents only a subtype of the broader transoral approach [6, 7]. Understanding the various transoral approaches and their associated outcomes is essential for informed surgical decision‐making when selecting the most appropriate technique. Therefore, this study aims to systematically review and synthesize the various transoral styloidectomy techniques, their advantages and disadvantages, and their associated outcomes. Our secondary aim is to introduce and describe an improved, endoscopic‐assisted transoral approach to styloidectomy.

## 2 Methods

**FIGURE 1:**
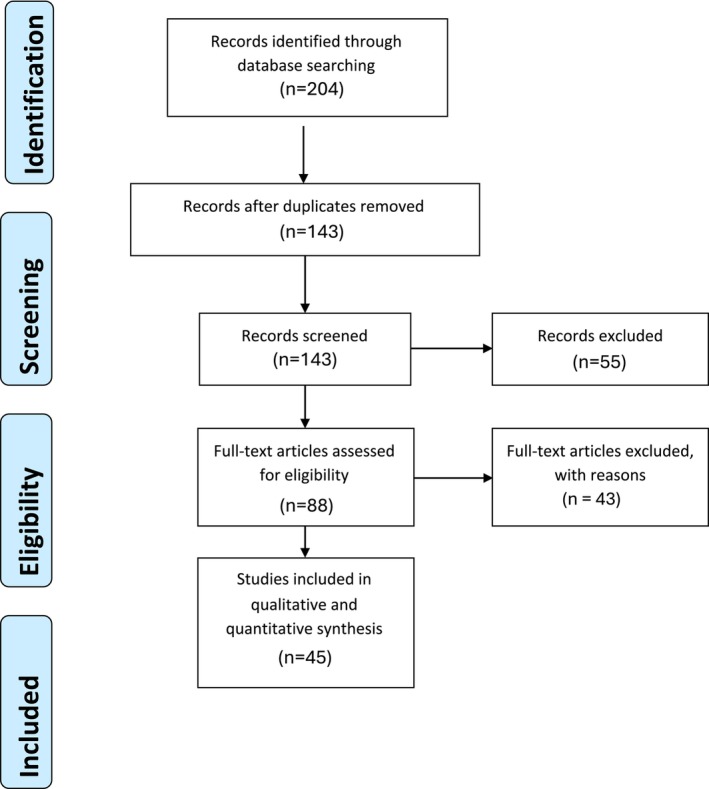
PRISMA flow diagram. [Color figure can be viewed at wileyonlinelibrary.com]

The PRISMA checklist (Figure 1) guided our study. A systematic search on PubMed, Embase, Cochrane Library, and Medline was conducted on November 11, 2025. We included studies on human subjects with Eagle's syndrome, either due to an elongated or calcified styloid process and/or stylohyoid ligament. Studies must have utilized a transoral approach and reported on surgical technique. Studies on extraoral techniques, cadaver studies, non‐English publications, and previous systematic reviews were excluded. The search methods for study identification can be found in the File S1. Two reviewers (NA and VH) independently screened in duplicate all titles, abstracts, and full texts of identified articles for eligibility and inclusion using the Rayyan screening tool. Disagreements in selection were reconciled by the principal investigator (C.H.S.) as needed. The final compiled list of articles underwent data extraction by two independent reviewers (N.‐H.A.‐R. and V.H.) using a standardized Excel spreadsheet. Data extraction focused on key study characteristics, patient demographics, type of transoral technique employed, laterality of resection (unilateral or bilateral), length of styloid resected, tonsillectomy‐sparing, relevant anatomic landmarks, intraoperative management of critical structures, duration of surgery, recovery time, surgical success rate (defined as complete resolution of symptoms), complication rate (intraoperative and postoperative), and follow‐up time. The quality of studies was assessed based on the methodological index for non‐randomized studies (MINORS) criteria (Table S1). A cut‐off score of > 12 was considered high quality, 8–12 as intermediate quality, and < 8 as low quality. Data was synthesized using narrative and tabular formats. Statistical analyses were conducted using STATA, version 18 (StataCorp, College Station, TX).

## 3 Results

Figure 1 displays our PRISM diagram. We initially identified 143 individual studies that were narrowed to 88 based on our exclusion criteria. After primary review, we excluded 43 articles, leaving 45 articles for in‐depth review and analysis. The descriptive statistics for the 45 studies can be found in Table 1. The earliest study was published in 2002, but most studies (*n* = 35) were published after 2014. The majority were single case reports (*n =* 25), and sample size ranged from 1 to 82 (median: 1, IQR: 1–11.5). Among the studies included, 100% were considered intermediate to high quality (MINOR score = 8–16; Table S1).

**TABLE 1:** Characteristics of included studies (*N* = 45).

Characteristic	Value (*N* = 45)
Year of publication	
Mode	2017
Range	2002–2025
Country of origin	
Bhutan	1 (2.22%)
Brazil	1 (2.22%)
China	3 (6.67%)
Colombia	1 (2.22%)
India	7 (15.56%)
Iran	1 (2.22%)
Iraq	1 (2.22%)
Italy	5 (11.11%)
Japan	2 (4.44%)
Korea	1 (2.22%)
Malaysia	3 (6.67%)
Nepal	2 (4.44%)
Poland	2 (4.44%)
Serbia	1 (2.22%)
Spain	1 (2.22%)
Turkey	4 (8.89%)
UK	1 (2.22%)
USA	8 (17.78%)
Study design	
Case report	25 (55.56%)
Case series	8 (17.78%)
Prospective cohort study	3 (6.67%)
Retrospective cohort study	7 (15.56%)
Cross‐sectional study	2 (4.44%)
Sample size	
Mean ± SD	9.09 ± 15.25
Median (IQR)	1 (1–11.5)
Range	1 to 82

### 3.1 Summary of Study Outcomes for Intraoral Approach

**FIGURE 2:**
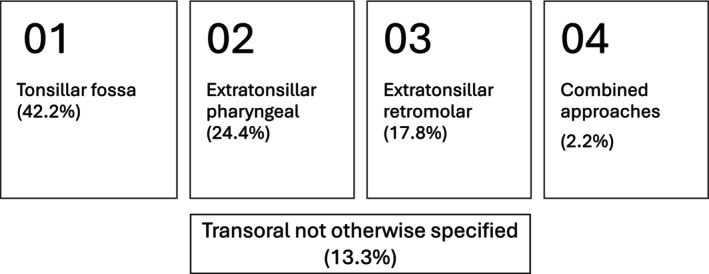
The four transoral approaches.

Table 2 provides a breakdown of the various transoral approaches and their outcomes. As shown in Figure 2, four approaches were described, including tonsillar fossa (42.2%), extratonsillar pharyngeal (24.4%), extratonsillar retromolar (17.8%), and combined (2.2%) approaches. Nine studies used visualization enhancing technologies, including robotic‐assisted [8, 9, 10], endoscopic‐assisted [11, 12, 13, 14, 15], microscopic‐assisted [16], and navigation‐assisted [17, 18]. Thirteen studies reported tonsillectomy‐sparing styloidectomy. Only one study [12] used nerve monitoring to monitor the facial nerve. No study reported on how critical vascular structures, such as ICA, were managed and no study used ICG fluorescence imaging to monitor vascular structures. The average length of resected styloid was 2.91 ± 1.33 cm (range: 1–6 cm). The average duration of surgery was 47 ± 22 min. The median follow‐up time was 6 months (IQR: 3–12). The overall pooled success rate for patients across the 45 studies was 94.1%.

**TABLE 2:** Characteristics of intraoral approach in studies reviewed (*N* = 45).

Surgical approach	Value (*N* = 45)
Tonsillar fossa	19 (42.2%)
Extratonsillar, pharyngeal	11 (24.4%)
Extratonsillar, retromolar	8 (17.8%)
Combined	1 (2.2%)
Transoral NOS^a^	6 (13.3%)
Visualization aid	
None	33 (75.00%)
Robotic‐assisted	3 (6.82%)
Endoscopic‐assisted	5 (11.36%)
Microscopic‐assisted	1 (2.27%)
Navigation‐assisted	2 (4.55%)
Laterality	
Unilateral	16 (35.56%)
Bilateral	23 (51.11%)
NOS	6 (13.33%)
Nerve monitoring used	1 (2.44%)
Tonsillectomy‐sparing	13 (31.71%)
Length of resected styloid (cm)	
Mean ± SD	2.91 ± 1.33
Median (IQR)	2.5 (2–3.8)
Duration of surgery (min)	
Mean ± SD	47 ± 22
Median (IQR)	43 (32–59)
Mean ± SD	26 ± 63
Median (IQR)	4 (1–16.5)
Follow‐up time (months)	
Mean ± SD	10.4 ± 15
Median (IQR)	6 (3–12)
Success rate^a^	
Mean ± SD	94.1%
Median (IQR)	100% (100%–100%)
Total sample size, *N*	407
Complications	
Intraoperative complications	0% (0%)
Postoperative complications, *n* (%)	25 (6.30%)

Abbreviations: IQR = interquartile range (Q1–Q3), NOS = not otherwise specified, SD = standard deviation.

^a^ Success rate defined as full symptom resolution.

Among the eight studies reporting a success rate below 100% [16, 19, 20, 21, 22, 23, 24, 25], two studies (2 patients) used a tonsillar fossa approach, four studies (27 patients) used an extratonsillar pharyngeal approach, one study (1 patient) used the retromolar approach, and one study (17 patients) did not report on the type of transoral approach used. Among studies with less than 100% success rate, only one study used a visual‐enhancing technique [16]. No study reported an intraoperative complication. Seven studies [10, 11, 20, 24, 25, 26, 27] reported postoperative complications including transient facial palsy, wound infection, wound dehiscence, facial artery pseudoaneurysm, bleeding/hematoma, surgical site numbness, first bite syndrome, trismus, dysphagia, moderate pain, and hypernasality or nasopharyngeal reflux. Of the seven studies reporting complications, one study used a tonsillar fossa approach [26], three used extratonsillar pharyngeal approach [11, 25, 27], one used extratonsillar retromolar approach [10], and two [20, 24] did not report on the type of approach used. Only two studies with postoperative complications used visual‐enhancing technique (robotic in [10] and endoscopic in [11]) (Table 3).

**TABLE 3:** Summary of findings.

References	Design	Country	Sample size	Indications^a^	Surgical approach	Visualization technique	Tonsillectomy sparing	Postoperative complication	Resected styloid (cm)	Success rate (%)	Recovery time (days)	Follow‐up time (months)
Aravindan et al. [28]	Case series	USA	2	Neural	Extratonsillar retromolar		Yes		3.00	100.00		12
Baharudin et al. [29]	Case report	Malaysia	1	Neural	Extratonsillar pharyngeal		No		2.00	100.00		24
Bareiss et al. [30]	Case Report	Turkey	1	Neural	Extratonsillar pharyngeal		No		2.00	100.00	0.25	3
Beder et al. [21]	Retrospective Clinical Trial	Brazil	19	Neural	Tonsillar fossa		NA		3.90	100.00		12
Beder et al. [31]	Case report	Iran	1	Neural	Tonsillar fossa		NA		4.25	100.00	270	9
Bedi et al. [32]	Case report	Malaysia	1	Neural	Extratonsillar retromolar		No		2.40	100.0	30	1
Caranti et al. [33]	Case series	Italy	7	Mixed	Unknown		NA		6.2	100		3
Cheng et al. [19]	Retrospective clinical study	USA	62	Neural	Tonsillar fossa		Yes		1.80	100.00	24	9
Dou et al. [17]	Prospective cohort	USA	12	Neural	Extratonsillar pharyngeal	Navigation	No		2.20	100.00		6
Ferretti [34]	Case report	Italy	1	Neural	Tonsillar fossa		No		3.5			4
Gallaway et al. [35]	Case report	UK	1	Neural	Extratonsillar pharyngeal		No		2.50	100.00		6
Hamamin et al. [36]	Case report	China	1	Neural	Tonsillar fossa		No		3.80	100.00		
Hardin et al. [20]	Retrospective cohort study	Japan	21	Mixed	Unknown		Yes	5 cases: first bite syndrome (some resolved), numbness (some resolved)	3.00	100.00	1	
Held et al. [26]	Retrospective case series	India	56	Mixed	Tonsillar fossa		No	5 cases of hypernasality or nasopharyngeal reflux (resolved with time), wound dehiscence, facial artery pseudoaneurysm (required embolization)		100.00		12
Hossein et al. [37]	Case report	India	1	Neural	Unknown		Yes			100.00		17
Jeong et al. [38]	Case report	Korea	1	Neural	Tonsillar fossa		No		5.00	100.00		0.25
Kadakia et al. [8]	case series	Italy	3	Mixed	Extratonsillar retromolar	Robotic				100.00	1	12
Al‐Abrar Ahmad Kailani et al. [5]	case report	Japan	1	Neural	Tonsillar fossa		Yes		1.75	100.00		
Kamil et al. [39]	Case report	Bhutan	1	Neural	Extratonsillar pharyngeal		No			100.00		0.25
Kapoor et al. [40]	Case series	Iraq	2	Neural	Extratonsillar retromolar		No		2.50	100.00		
Kapoor et al. [16]	prospective study	Malaysia	25	Neural	Tonsillar fossa	Microscopic	No		2.15	100.00		0.25
Kiralj et al. [41]	Case series	USA	2	Neural	Unknown		Yes		2.00	100.00		1
Kumai et al. [22]	Retrospective	India	14	Neural	Tonsillar fossa		No			100.00		15
Leming et al. [12]	Case report	India	1	Neural	Extratonsillar retromolar	Endoscopic	Yes			100.00	0.25	0.25
Liu et al. [42]	Case report	Serbia	1	Neural	Combined		NA		2.00	100.00	3	
Masalski et al. [13]	Case report	Poland	1	Mixed	Tonsillar fossa	Endoscopic	No		4.01	100	14	5
Meenakshisundaram et al. [43]	Case report	India	1	Neural	Tonsillar fossa		NA		2.92	100.00	14	12
Mevio et al. [14]	Case report	USA	1	Neural	Tonsillar fossa	Endoscopic	Yes		1.00	100.00	1	
Montevecchi et al. [9]	Case report	Poland	1	Neural	Extratonsillar retromolar	Robotic	Yes		1.65	100.00	120	12
Müderris et al. [44]	Retrospective case series	Spain	8	Neural	Tonsillar fossa					100.00	1	
Pokharel et al. [45]	Prospective, analytical study	Saudi Arabia	39	Neural	Tonsillar fossa		Yes			100.00	26.5	
Pradhan et al. [46]	Case report	Turkey	1	Neural	Tonsillar fossa		No		1.50	100.00	0.17	20
Regmi et al. [27]	Cross sectional	Colombia	24	Neural	Extratonsillar pharyngeal			3 cases: moderate pain, trismus, dysphagia (resolved), wound dehiscence (healed secondarily)	3.60	90.00		84
Rizzo‐Riera et al. [10]	Case series	China	6	Neural	Extratonsillar retromolar	Robotic	Yes	1 case: suture dehiscence (healed secondarily)	2.00	100.00	2.06	3
De Souza Carvalho et al. [47]	Case report	Japan	1	Neural	Extratonsillar pharyngeal	Navigation‐assisted	No		1.90	83.00	5	
Subramaniam et al. [48]	Case report	Turkey	1	Neural	Extratonsillar pharyngeal				2.25	76.04		6
Sukegawa et al. [18]	Case report	Turkey	1	Neural	Tonsillar fossa		No		6.00	65.00	0.25	12
Terenzi et al. [11]	Case report	USA	1	Neural	Extratonsillar pharyngeal	Endoscopic		1 case: transient (12 h) right facial palsy (resolved spontaneously)	1.00	62.00		3
Torres et al. [23]	case series	Nepal	11	Neural	Extratonsillar pharyngeal		Yes			100.00		6
Usaklioglu et al. [24]	retrospective case series	India	17	Neural	Unknown		No	7 cases: wound infection, mild first bite syndrome (transient), numbness in the surgical site (transient).		50.00		3
Waclawek et al. [49]	Case series	Nepal	5	Neural	Unknown		No			100.00	7	
Walli et al. [50]	Case report	USA	1	Neural	Tonsillar fossa		No		3.50	80.00		1.8
Walters et al. [25]	Cross sectional	USA	47	Mixed	Extratonsillar pharyngeal		Yes	3 cases: wound dehiscence, postop bleeding/hematoma		100.00		
Al Weteid and Miloro [51]	Case report	China	1	Neural	Extratonsillar retromolar		No		3.90	70.00	6	36
Yadav et al. [15]	Case report	India	1	Neural	Tonsillar fossa	Endoscopic	No		5	100		3

Abbreviation: NA = not mentioned.

^a^ Neural indications involve symptoms of pain (e.g., odynophagia, otalgia, etc.) whereas vascular indications include internal cortaid artery or internal jugular vein compression (e.g., dizziness, syncope, pulsatile tinnitus, etc.).

### 3.2 Enhanced Endoscopic Approach: Extratonsillar Retromolar

**FIGURE 3:**
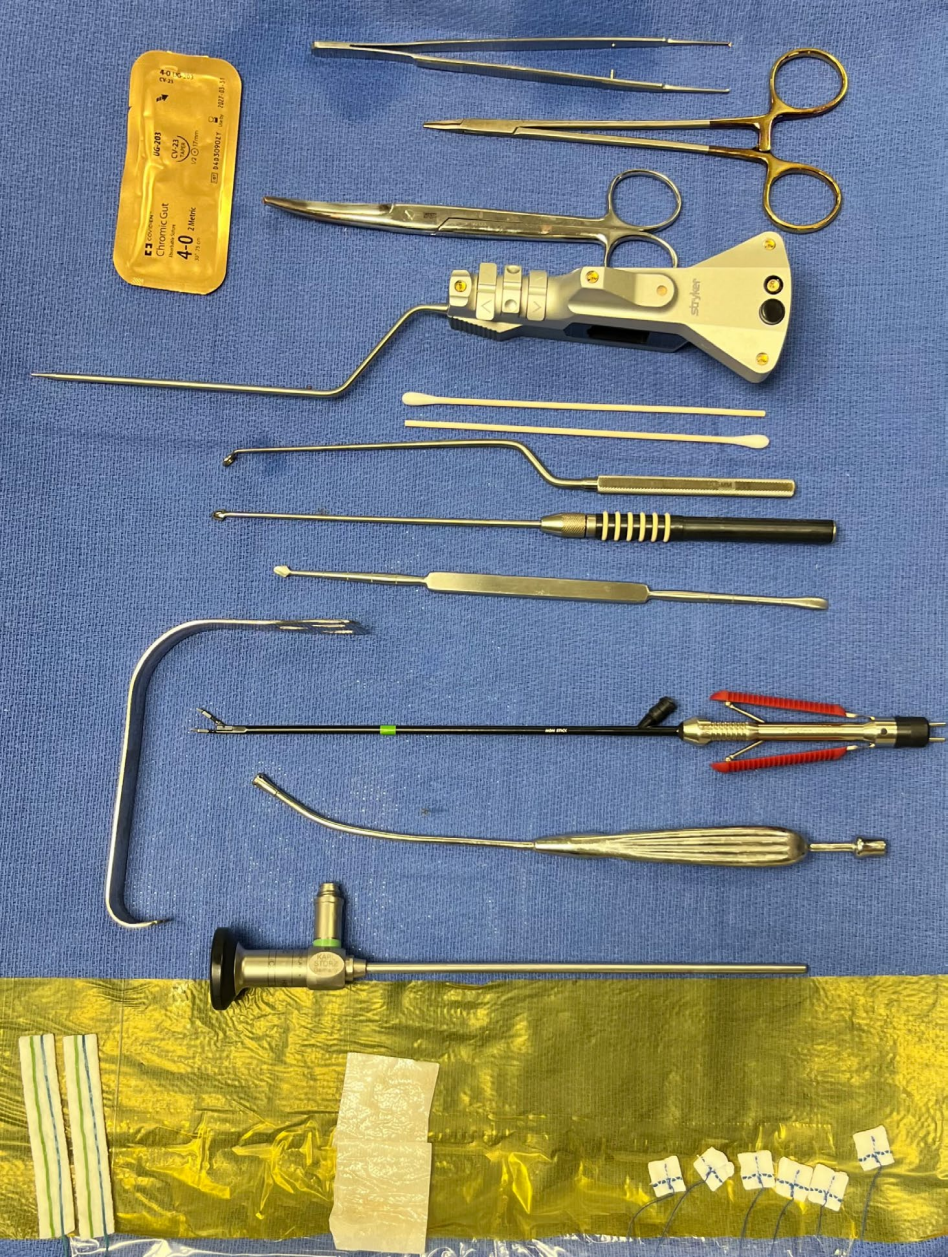
Surgical tools used during the enhanced endoscopic extratonsillar–retromolar styloidectomy approach. [Color figure can be viewed at wileyonlinelibrary.com]

A 46‐year‐old female presented with right‐sided stabbing head/neck pain exacerbated by head movement and swallowing. CT imaging demonstrated an elongated right styloid process, confirming a diagnosis of Eagle's syndrome. A right‐sided styloidectomy was performed via tonsil‐sparing, endoscopic‐assisted transoral approach. A mucosal incision between the ramus of the mandible and the maxillary tuberosity was extended inferiorly across the retromolar trigone to the lateral limit of the anterior tonsillar pillar. A 0‐degree endoscope was used to visualize the oropharynx. Blunt dissection was used to separate the muscles, allowing palpation of the elongated styloid process just medial to the ramus of the mandible. Neuromonitoring with somatosensory evoked potentials (SSEP) was used due to the risk of ICA injury. Doppler and ICG fluoroscopy were used to confirm the location of the ICA. A Kerrison rongeur was used to transect the styloid process as high as possible, removing approximately 1.5 cm segment of bone (Figure 3). The incision was closed with interrupted 4–0 chromic sutures. There were no intraoperative complications (Video S1). At 3 months follow‐up, complete resolution of symptoms was noted.

## 4 Discussion

### 4.1 Key Findings

In this study, we systematically and comprehensively reviewed the existing literature on transoral styloidectomy. Our review included 45 studies encompassing 407 patients. Indications for a transoral styloidectomy include a constellation of symptoms consistent with Eagles's syndrome, exclusion of other pathology, a palpable styloid process in the tonsillar fossa or lateral pharyngeal wall that reproduces the patient's symptoms, and avoidance of morbidity associated with an external approach [51]. All studies involved patients reporting some level of neural symptoms as the indication for surgery (odynophagia, otalgia, neck pain, etc.) and only six studies had patients with neural and vascular symptoms (i.e., dizziness, syncope, pulsatile tinnitus, etc.). The transoral approach allows distal dissection of a calcified stylohyoid ligament. However, attempting full resection up to the mastoid is often impractical, carries significant risk, and is generally unnecessary. In most cases, symptom relief does not require complete excision of the styloid process to the skull base [26]. Contraindications to the transoral approach include limited mouth opening resulting in poor surgical access, proximity to major neurovascular structures, active oral or neck infections, inability to tolerate general anesthesia, and a poor surgical candidate [51].

Historically, the transoral approach was limited by inadequate surgical exposure and visualization of the styloid and its associated structures. However, this review demonstrates that the integration of visual‐enhancing techniques, such as image‐based navigation, along with endoscopic, microscopic, and robotic‐assisted methods has effectively mitigated this limitation. The majority of studies (37 studies involving 360 patients) reported a 100% success rate, while eight studies reported a success rate ranging from 50% to 90% (47 patients). Of the studies with less than 100% success rate, only one utilized a visual‐enhancing technique—the microscopic‐assisted tonsillar fossa approach. No study reported intraoperative complications, and among the seven studies with postoperative complications, only two used visual‐enhancing techniques (a robotic‐assisted extratonsillar retromolar approach and an endoscopic‐assisted extratonsillar pharyngeal approach); however, these complications were transient and not clearly associated with variations in technique. None of the seven studies reporting postoperative complications, including one patient with transient facial nerve injury, incorporated nerve monitoring. Finally, our case demonstrates that the endoscopic approach can be further optimized with neuromonitoring, Doppler sonography, and ICG fluoroscopy to prevent injury to critical vascular structures.

### 4.2 Advancement in the Transoral Approach

The styloid process originates from the temporal bone, projects inferiorly and medially towards the pharynx, where it inserts onto the lesser horn of the hyoid bone by the fibrous stylohyoid ligament [3]. Several critical neurovascular structures travel along the length of the styloid process and stylohyoid ligament, including the ICA, internal jugular vein, and multiple cranial nerves—the facial, glossopharyngeal, vagus, and hypoglossal nerves [52]. Given its proximity to these critical structures, minimizing surgical morbidity is of utmost importance when selecting a styloidectomy approach. The intraoral approach is a minimally invasive technique that provides safer and more direct access to the styloid process while minimizing injury to healthy tissue [19, 28, 29, 42]. Compared to the extraoral approach, it avoids extensive soft tissue dissection, eliminates visible external scarring, reduces surgical morbidity, offers shorter operative and recovery time, and demonstrates improved outcomes [11, 20, 27, 32, 35].

The risk of neurovascular injury during a transoral approach can be minimized by a visual‐enhancing technique augmented with intraoperative neurovascular monitoring such as Doppler ultrasound, ICG fluoroscopy, and nerve monitoring. Among the studies reviewed, only one used nerve monitoring to monitor the facial nerve in a pediatric patient presenting with bilateral facial pain [12]. Nerve monitoring is particularly beneficial in patients with classic‐type Eagle's syndrome, which presents as face, ear, throat, neck, and jaw pain due to compression of sensory nerves [1, 3]. In the case of carotid‐type Eagle's syndrome, which presents as dizziness, vertigo, syncope, visual changes, transient ischemic attacks, or stroke due to compression of the carotid artery, augmenting a visual‐enhancing technique with Doppler and ICG fluoroscopy can further reduce the risk of vascular injury [3, 51]. Vascular injury is a rare but significant risk of the transoral styloidectomy due to the proximity to the ICA and IJV [53]. Therefore, neuromonitoring is recommended in high‐risk surgeries, especially those with vascular compression. Furthermore, among the studies reviewed, one study involving 62 patients reported the use of coblation during an intraoral approach [19]. In that study, coblation was associated with superior hemostasis, decreased bleeding, shorter operative time, less tissue injury, and faster recovery time [19]. Additionally, a tonsillectomy‐sparing technique can further reduce procedure time, avoid the additional morbidity of tonsillectomy, and decrease postoperative pain [8, 11, 40, 54].

## 5 Implications for Clinical Practice

This systematic review focused on transoral styloidectomy and its various surgical techniques. By synthesizing data from 41 studies encompassing 407 patients, this study provides a robust review of operative techniques, complication profiles, and surgical outcomes. We highlight specific strategies that can enhance surgical safety and outcomes in both the classic and vascular‐type Eagle's syndrome. Notably, our study highlights evolving innovations to the transoral approach and offers insight into the benefits of the tonsillectomy‐sparing technique and neurovascular monitoring. Our results provide valuable guidance for surgeons considering the transoral styloidectomy approach and support its safety and efficacy. Ultimately, the selection of the surgical approach depends on both surgeon and patient preference. However, we provide evidence that the transoral approach is safe; when augmented with visual‐enhancing techniques and neurovascular monitoring, it can achieve excellent outcomes, including high success rates and minimal complications.

While a comprehensive list of search terms was used to conduct our literature search, there is a possibility that some relevant studies were missed. Most of the studies included in the review are limited to case reports, small case series, and retrospective cohort studies, which inherently limits the quality of evidence. Common limitations of the studies include the lack of randomization and blinding, selection bias, and non‐prospective study design. Notably, only a small number of studies used visual‐enhancing and neurovascular monitoring techniques, limiting our ability to vigorously compare outcomes and draw definitive conclusions using metanalyses. While augmented techniques may improve surgical outcomes, accessibility is limited based on available resources and associated costs. Furthermore, the use of neuromonitoring in our case report was limited to detection of cerebral ischemia and did not include electromyography of motor cranial nerves. Additional research is needed to demonstrate the added value of enhanced visualization and neurophysiologic monitoring. Comparison of techniques would be enhanced by standardized reporting of outcomes. Larger prospective studies are needed to compare outcomes of the enhanced endoscopic extratonsillar retromolar approach with traditional transoral techniques.

The transoral approach allows distal dissection of a calcified stylohyoid ligament; however, additional data are needed to better define the proximal and distal limits of dissection for the various surgical approaches. Lastly, there may be selection bias in the choice of surgical approach: patients with shorter styloid process may be selected for a transoral approach, whereas those with a longer styloid process are selected for an extraoral (transcervical) approach. Data is lacking regarding the extent of styloidectomy necessary to resolve symptoms and the limitations of proximal and distal stylohyoid access with various approaches.

## 6 Conclusions

Based on intermediate to high quality evidence, this systematic review suggests that transoral styloidectomy is a safe and effective surgical approach for the treatment of Eagle's syndrome, with high success rates and low complication rates. Surgeons considering the transoral approach should recognize that incorporating a visual‐enhancing technique may further improve success rates and decrease the risk of complications. Augmenting the intraoral approach with neurovascular monitoring can further improve patient safety.

## Author Contributions

**Nana‐Hawwa Abdul‐Rahman:** conceptualization and design, data curation, formal statistical analysis, investigation, methodology, supervision, validation, writing – original draft, writing – review and editing. **Vanessa Helou:** data curation. **Lauren A. Gardiner:** data curation. **Paul A. Gardner:** conceptualization and design, methodology, writing – review and editing. **Carl H. Snyderman:** conceptualization and design, investigation, methodology, supervision, validation, writing – original draft, writing – review and editing.

## Funding

The authors have nothing to report.

## Conflicts of Interest

The authors declare no conflicts of interest.

## Supporting information


**Table S1:** Risk of bias assessment (*N* = 45), per methodological index for non‐randomized studies (MINORS) criteria.

**Video S1:** Enhanced endoscopic approach: extratonsillar retromolar.

**File S1:** Search strategy.

## Data Availability

The data that support the findings of this study are available from the corresponding author upon reasonable request.
