# Supplementary material for: Transoral Styloidectomy Approach: A Systematic Review and Enhanced Endoscopic Approach
Source: Head Neck. 2026 Jan 16;48(6):1567–78. doi: 10.1002/hed.70170 (PMC13155192; doi:10.1002/hed.70170)
Supplement: Supplementary file 1 — Table S1: Risk of bias assessment (N = 45), per methodological index for non‐randomized studies (MINORS) criteria. [file HED-48-1567-s003.docx]

**Supplementary Table 1. Risk of bias assessment (*N*=45), per Methodological Index for Non-randomized studies (MINORS) criteria.**

|  | *MINORS criteria* | | | | | | | |  |  |
| --- | --- | --- | --- | --- | --- | --- | --- | --- | --- | --- |
| Study  Author (Year) | Q1 | Q2 | Q3 | Q4 | Q5 | Q6 | Q7 | Q8 | total | Quality |
| Aravindan 2023 | 2 | 1 | 2 | 2 | 2 | 2 | 2 | 0 | 13 | High |
| Baharudin 2012 | 2 | 0 | 2 | 2 | 2 | 2 | 2 | 0 | 12 | Intermediate |
| Bareiss 2017 | 2 | 0 | 2 | 2 | 2 | 2 | 2 | 0 | 12 | Intermediate |
| Beder 2005 | 2 | 2 | 0 | 2 | 2 | 2 | 1 | 0 | 11 | Intermediate |
| Beder 2006 | 2 | 0 | 2 | 2 | 2 | 2 | 2 | 0 | 12 | Intermediate |
| Bedi 2019 | 1 | 0 | 2 | 2 | 2 | 2 | 2 | 0 | 11 | Intermediate |
| Caranti 2024 | 2 | 2 | 0 | 2 | 2 | 2 | 2 | 0 | 12 | Intermediate |
| Cheng 2017 | 2 | 2 | 0 | 2 | 2 | 2 | 2 | 0 | 12 | Intermediate |
| Dou 2016 | 2 | 2 | 2 | 2 | 2 | 2 | 2 | 0 | 14 | High |
| Ferretti 2025 | 2 | 0 | 2 | 2 | 2 | 2 | 2 | 0 | 12 | Intermediate |
| Gallaway 2017 | 2 | 0 | 2 | 2 | 2 | 2 | 2 | 0 | 12 | Intermediate |
| Hamamin 2023 | 2 | 0 | 2 | 2 | 2 | 2 | 2 | 0 | 12 | Intermediate |
| Hardin 2018 | 2 | 2 | 0 | 2 | 2 | 2 | 2 | 0 | 12 | Intermediate |
| Held 2024 | 2 | 2 | 0 | 2 | 2 | 2 | 0 | 0 | 10 | Intermediate |
| Hossein 2010 | 0 | 0 | 2 | 2 | 2 | 2 | 2 | 0 | 10 | Intermediate |
| Jeong 2021 | 1 | 0 | 2 | 2 | 2 | 2 | 2 | 0 | 11 | Intermediate |
| Kadakia 2018 | 2 | 2 | 2 | 1 | 1 | 2 | 2 | 0 | 12 | Intermediate |
| Kailani 2023 | 2 | 0 | 2 | 2 | 2 | 2 | 2 | 0 | 12 | Intermediate |
| Kamil 2015 | 2 | 0 | 2 | 2 | 2 | 2 | 2 | 0 | 12 | Intermediate |
| Kapoor 2015 | 2 | 2 | 2 | 1 | 2 | 2 | 2 | 0 | 13 | High |
| Kapoor 2024 | 2 | 2 | 2 | 2 | 2 | 2 | 1 | 0 | 13 | High |
| Kiralj 2015 | 1 | 2 | 2 | 1 | 1 | 2 | 2 | 0 | 11 | Intermediate |
| Kumai 2016 | 2 | 2 | 0 | 2 | 2 | 2 | 2 | 0 | 12 | Intermediate |
| Leming 2024 | 2 | 0 | 0 | 2 | 2 | 2 | 2 | 0 | 10 | Intermediate |
| Liu 2017 | 2 | 0 | 0 | 1 | 1 | 2 | 2 | 0 | 8 | Intermediate |
| Masalski 2025 | 2 | 0 | 2 | 2 | 2 | 2 | 2 | 0 | 12 | Intermediate |
| Meenakshisundaram 2024 | 2 | 0 | 2 | 2 | 2 | 2 | 2 | 0 | 12 | Intermediate |
| Mevio 2021 | 2 | 0 | 2 | 2 | 2 | 2 | 2 | 0 | 12 | Intermediate |
| Montevecchi 2019 | 2 | 0 | 2 | 2 | 2 | 2 | 2 | 0 | 12 | Intermediate |
| Muderris 2014 | 2 | 2 | 2 | 2 | 2 | 2 | 2 | 0 | 14 | High |
| Pokharel 2015 | 2 | 2 | 2 | 2 | 1 | 2 | 2 | 0 | 13 | High |
| Pradhan 2022 | 2 | 0 | 2 | 2 | 2 | 2 | 2 | 0 | 12 | Intermediate |
| Regmi 2021 | 2 | 2 | 1 | 1 | 1 | 2 | 2 | 2 | 13 | High |
| Rizzo-Riera 2020 | 2 | 2 | 1 | 1 | 1 | 2 | 2 | 0 | 11 | Intermediate |
| Souza Carvalho 2009 | 2 | 0 | 2 | 1 | 1 | 2 | 2 | 0 | 10 | Intermediate |
| Subramaniam 2002 | 2 | 0 | 2 | 2 | 2 | 2 | 2 | 0 | 12 | Intermediate |
| Skuegawa 2017 | 1 | 0 | 2 | 2 | 2 | 2 | 2 | 0 | 11 | Intermediate |
| Terenzi 2019 | 2 | 0 | 2 | 2 | 2 | 2 | 2 | 0 | 12 | Intermediate |
| Torres 2014 | 2 | 2 | 2 | 2 | 2 | 2 | 2 | 0 | 14 | High |
| Usaklioglu 2021 | 2 | 2 | 0 | 2 | 2 | 2 | 2 | 0 | 12 | Intermediate |
| Waclawek 2020 | 2 | 1 | 2 | 2 | 2 | 2 | 2 | 0 | 13 | High |
| Walli 2018 | 2 | 0 | 2 | 2 | 2 | 2 | 2 | 0 | 12 | Intermediate |
| Walters 2023 | 2 | 2 | 0 | 2 | 2 | 2 | 2 | 0 | 12 | Intermediate |
| Weteid 2015 | 2 | 0 | 2 | 2 | 2 | 2 | 2 | 0 | 12 | Intermediate |
| Yadav 2025 | 2 | 0 | 2 | 2 | 2 | 2 | 2 | 0 | 12 | Intermediate |

Q1: A clearly stated aim

Q2: Inclusion of consecutive samples

Q3: Prospective collection of data

Q4: Endpoints appropriate to the aim of the study

Q5: Unbiased assessment of the study endpoint

Q6: Assessment tests appropriate with the aim

Q7: Loss of samples <5%

Q8: Prospective calculation of the study size

Rating per question: 0=not reported, 1=reported but inadequate, 2=reported and adequate, maximum score of 16

Score quality: > 12: high, 8-12: intermediate, <8: low
