# Supplementary material for: Transoral Styloidectomy Approach: A Systematic Review and Enhanced Endoscopic Approach
Source: Head Neck. 2026 Jan 16;48(6):1567–78. doi: 10.1002/hed.70170 (PMC13155192; doi:10.1002/hed.70170)
Supplement: Supplementary file 3 — File S1: Search strategy. [file HED-48-1567-s002.docx]

**Search Strategies** (November 11, 2025)

**PubeMed**

(("Eagle Syndrome"[Mesh] OR "eagle syndrome"[TIAB] OR "styloidectomy"[TIAB]) AND ("surgical approach"[TIAB] OR "transoral approach"[TIAB] OR "cervical approach"[TIAB] OR "intraoral approach"[TIAB] OR "endoscopic"[TIAB] OR "robotic"[TIAB] OR "Transoral Styloidectomy”)) **65**

**Medline**

Ovid MEDLINE(R) Epub Ahead of Print and In-Process, In-Data-Review & Other Non-Indexed Citations and Daily <November 11, 2025>

1 eagle syndrome.mp. 511

2 styloid process.mp. 1336

3 1 or 2 1520

4 styloidectomy.mp. 206

5 endoscopic styloidectomy.mp. 0

6 4 or 5 206

7 transoral.mp. 6482

8 transoral approach.mp. 1010

9 endoscopy.mp. or exp Endoscopy/ 467327

10 surgical approach.mp. 38386

11 surgical technique.mp. 48676

12 exp Minimally Invasive Surgical Procedures/ 632385

13 minimally invasive surgery.mp. 18592

14 intraoral approach.mp. 734

15 exp Robotic Surgical Procedures/ or robotic surgery.mp. 25451

16 transoral robotic surgery.mp. 1262

19 7 or 8 or 9 or 10 or 11 or 12 or 13 or 14 or 15 or 16 771596

20 13 and 15 and 19 **34**

**Embase**

('eagle syndrome'/exp OR 'eagle syndrome') AND ('styloidectomy'/exp OR 'styloidectomy' OR 'styloid process') AND ('transoral approach' OR 'transoral' OR 'robotic surgery'/exp OR 'robotic' OR 'endoscopic' OR 'endoscopic surgery'/exp OR 'surgical approach' OR 'surgical technique' OR 'minimally invasive') **107**
